# Generalized Retinal Artery/Vein Segmentation via Multi-Dataset Fine-Tuning and Pathology Subgroup Analysis

**DOI:** 10.3390/bioengineering13080882

**Published:** 2026-07-30

**Authors:** Seo Gyeong Lee, Ju-Hyuck Han

**Affiliations:** Department of Medical IT Engineering, Konyang University, 158 Gwanjeodong-ro, Seo-gu, Daejeon 35365, Republic of Korea; 23615038@konyang.ac.kr

**Keywords:** artery–vein classification, color fundus photography, convolutional neural networks, deep learning, domain generalization, image segmentation, retinal imaging

## Abstract

Background: Automatic artery/vein (A/V) segmentation in color fundus photography underpins retinal biomarkers such as the arteriolar-to-venular ratio (AVR), yet single-dataset models generalize poorly across institutions and pathologies. Methods: Using pre-trained Recursive Refinement W-Net (RRWNet) weights as initialization, eight heterogeneous datasets were jointly fine-tuned under a single set of weights for 50 epochs: five A/V-labeled (Fundus-AVSeg, LES-AV, RITE, FIREFLY-Gen, PSEUDO_AV) and three vessel-only (DRIVE, CHASE_DB1, HRF). A vessel-only loss separation strategy applied only the vessel-channel loss to vessel-only datasets, preventing A/V metric dilution; post-processing combined field-of-view masking, connected-component denoising, and morphological refinement, outputting artery, vein, and vessel masks with crossing and junction maps. Results: Loss separation recovered validation DSC from 0.518 to 0.570 (AUC up to 0.959). On re-inference across four datasets (332 images), the single-weight model attained mean-AV DSC 0.657 ± 0.085 and AUC 0.976 ± 0.016; across normal, AMD, glaucoma, and DR subgroups, performance was statistically indistinguishable (Kruskal–Wallis, all *p* > 0.36), indicating consistent behavior across pathologies. Conclusions: Within a leakage-aware internal evaluation, the single-weight model produced stable A/V segmentation across the four datasets and across pathology subgroups, indicating preliminary internal consistency rather than proven cross-domain generalization. Because the evaluation reuses data seen during training and no external or patient-level held-out set was used, robustness claims are deferred; leakage-free external validation and patient-level re-evaluation are identified as the essential next steps.

## 1. Introduction

The retinal vasculature is the only microvascular system in the human body amenable to direct, non-invasive observation. Color fundus photography enables quantitative analysis of artery and vein (A/V) diameters, trajectories, and branching patterns [1]. Among the derived measures, the arteriolar-to-venular ratio (AVR) has been reported as an imaging biomarker statistically associated with the onset and progression of major ophthalmic and systemic diseases, including hypertension, diabetic retinopathy (DR), glaucoma, and age-related macular degeneration (AMD) [2]. In particular, Niemeijer et al. [3] proposed a system for automated measurement of the arteriolar-to-venular diameter ratio from color fundus photographs and quantitatively demonstrated the feasibility of automated AVR computation in IEEE Trans. Med. Imaging. Accurate AVR computation requires pixel-level A/V segmentation with topological consistency—that is, each vessel must be labeled with the same class from its origin to its terminus. However, manual labeling of a single fundus image requires tens of minutes, making clinical workflow integration impractical, and thus driving strong clinical demand for automated A/V segmentation [4].

Prior to deep learning, segmentation relied on hand-crafted features: Soares et al. [5] fed 2D Gabor wavelet responses into a classifier, and Mendonça and Campilho [6] combined centerline detection with morphological reconstruction. Beyond retinal imaging, transfer learning and multi-source deep-learning frameworks have been applied across diverse biomedical image domains, including knee-joint MRI analysis [7] and stem-cell-derived endothelial-cell identification in photomicrographs [8], illustrating the broad applicability of cross-domain fine-tuning and data-driven fusion in heterogeneous medical imaging. After deep neural networks were introduced, patch-based DNNs by Liskowski and Krawiec [9], a fully connected CRF model by Orlando et al. [10], and segment-level/pixel-wise combined losses by Yan et al. [11] were reported successively in IEEE Trans. Med. Imaging and IEEE Trans. Biomed. Eng., rapidly advancing segmentation accuracy. Even within the classical paradigm, Zhang et al. [12] further boosted robustness by using locally adaptive derivative frames in orientation-score space. Focusing specifically on A/V classification, Dashtbozorg et al. [13] proposed an automatic graph-based approach, Estrada et al. [14] performed classification via topology estimation, and Galdran et al. [15] introduced an uncertainty-aware formulation; these studies established the methodological foundation on which later end-to-end deep models were built. Building on this, deep-learning-based retinal vessel segmentation has evolved primarily around U-Net [16]-family FCNNs, with recent efforts pushing beyond semantic segmentation to directly learning topological consistency. Karlsson and Hardarson [17] stacked four U-Net-style networks in series and achieved 95.6% artery accuracy on RITE. Galdran et al. [18] proposed a W-Net cascading two lightweight U-Nets. Morano et al. [19] extended this into RRWNet, combining a base segmentation network with a recursive refinement network that iteratively refines only the A/V maps without re-inputting the original image, effectively reducing manifest errors where a vessel label flips mid-course. Meanwhile, Go et al. [20] used a latent diffusion model to generate synthetic fundus images paired with A/V masks, demonstrating the potential of synthetic augmentation to supplement segmentation training under data-scarce conditions.

Despite these advances, four common limitations obstruct clinical deployment. First, state-of-the-art models [17,18,19] are mostly trained and evaluated on a single public dataset—RITE [21], LES-AV [22], or HRF [23]—and suffer sharp performance drops when the domain shifts to different institutions, devices, or patient populations. Second, public A/V datasets are predominantly composed of normal or mild DR cases, leaving model performance on clinically common AMD, glaucoma, and severe DR images unvalidated. Third, A/V-labeled public datasets contain only 22–100 images each, while vessel-only-label datasets (DRIVE [24], CHASE_DB1 [25], HRF [23]) are comparatively abundant yet difficult to exploit within the same training pipeline due to label heterogeneity, limiting overall data utilization. Fourth, existing evaluations focus mainly on accuracy and area under the curve (AUC), leaving positive predictive value (PPV) and other false-positive-suppression metrics underreported, and subgroup analyses quantifying performance variation by pathology type are almost entirely absent.

This study proposes a generalized A/V segmentation framework applicable to multi-domain and multi-pathology fundus images with a single model. Using pre-trained RRWNet [19] weights as initialization, eight heterogeneous datasets are jointly fine-tuned under a single set of weights: five A/V-labeled datasets (Fundus-AVSeg [26], LES-AV [22], RITE [21], FIREFLY-Gen [20], PSEUDO_AV) and three vessel-only-label datasets (DRIVE [24], CHASE_DB1 [25], HRF [23]). To prevent A/V metric dilution arising from label heterogeneity, a vessel-only loss separation strategy is introduced, backpropagating only the vessel-channel loss for vessel-only datasets. At inference, post-processing combines field-of-view (FOV) masking, connected-component (CC) noise removal, and morphological refinement, while simultaneously outputting artery, vein, and vessel masks alongside crossing and junction maps to provide the foundation for downstream quantitative analysis.

The contributions of this paper are summarized as follows.

- We present an integrated fine-tuning framework that jointly trains a single model on eight heterogeneous datasets with real, synthetic, and vessel-only labels. Relative to single-dataset-dedicated training [19], we report the internal consistency behavior of a single-weight model across four datasets (332 images); because this is a re-inference (leakage-aware) evaluation rather than external validation, we do not claim generalization superiority.
- We introduce a vessel-only loss separation strategy that mitigates A/V metric dilution caused by label heterogeneity and report the quantitative effect: validation DSC recovers from 0.518 to 0.570, and AUC reaches its highest value of 0.959.
- We conduct a four-pathology subgroup analysis (normal, AMD, glaucoma, DR) and quantitatively compare per-pathology performance differences using the same metrics: an exploratory Kruskal–Wallis test shows no significant difference among the four subgroups (all *p* > 0.36), consistent with pathology-robust behavior.
- We construct an 8-channel output pipeline that simultaneously produces artery, vein, and vessel masks together with crossing and junction maps, providing the basis for automated computation of clinical quantitative indices such as AVR and vessel complexity.

The remainder of this paper is organized as follows. Section 2 reviews related work on retinal A/V segmentation and synthetic data. Section 3 describes the methodology, including model architecture, dataset integration, the loss separation strategy, post-processing, and the experimental setup. Section 4 reports per-dataset and per-pathology performance results along with qualitative findings. Section 5 discusses trade-offs, PPV patterns, limitations, and clinical implications. Section 6 concludes with future directions.

## 2. Related Work

### 2.1. RRWNet-Based Recursive Refinement

RRWNet, proposed by Morano et al. [19], is a W-shaped architecture that serially connects two U-Net-style FCNNs. The first network, FCNN_1_, serves as the base subnetwork and simultaneously produces coarse segmentation maps for artery (A), vein (V), and vessel (BV) from an RGB fundus image. The second network, FCNN_2_, is the recursive refinement subnetwork and is applied *K* times iteratively; at each iteration, it receives only the A/V segmentation maps from the previous step, without re-inputting the original image. This design forces the refinement network to learn the topological structure of the segmentation maps themselves, reducing manifest classification errors in which the same vessel erroneously switches from artery to vein mid-course, and yielding A/V accuracy of 96.66% with COR improvement of +12.58 pp [19].

### 2.2. Synthetic Retinal Image Generation

Go et al. [20] proposed a three-stage pipeline: generating masks with an A/V-mask-conditioned DDPM (GM stage), synthesizing fundus images with pix2pixHD (GI stage), and upscaling with EDSR (GE stage). The vessel-weighted loss directly inspired the vessel-only loss separation of this study. The photorealism of the synthesized images was validated with a Turing test accuracy of 41.25% [20]. The synthesized fundus images produced by this pipeline are referred to as the FIREFLY-Gen dataset [20] in this study, and 12,000 images in total (6000 low-resolution and 6000 high-resolution) are included in training.

### 2.3. Common Limitations of Existing A/V Segmentation

State-of-the-art A/V segmentation studies rely on single-dataset-dedicated training. Karlsson and Hardarson [17] achieved 95.6% artery accuracy on RITE with four serially stacked U-Nets yet required separate hyperparameter searches for each dataset. Chen et al. [27] reported RITE mAV DSC of 0.963 with TW-GAN using a topology-aware loss, and Morano et al. [19] also trained separate weights for RITE [21], LES-AV [22], and HRF [15] individually. While single-dataset-dedicated training achieves peak performance within its training domain, performance drops sharply when the model is transferred to images from different institutions, devices, or patient populations—a finding widely reported in the literature. Furthermore, since public A/V datasets are predominantly composed of normal or mild DR cases, performance on AMD, glaucoma, and severe DR images has not been separately validated.

### 2.4. Distinction of This Study

This study directly addresses these limitations and differentiates itself as follows. (i) Unlike prior work that uses only one of RITE [21], LES-AV [22], or HRF [15], eight heterogeneous datasets are jointly trained under a single set of weights. (ii) A vessel-only loss separation strategy is introduced to handle label heterogeneity. (iii) Segmentation performance is separately quantified across four pathology subgroups: normal, AMD, glaucoma, and DR. (iv) Five metrics—DSC, sensitivity (Se), specificity (Sp), PPV, and AUC—are reported for each of the artery, vein, and vessel channels, making false-positive patterns visible.

## 3. Materials and Methods

### 3.1. Backbone Model—RRWNet

**Figure 1:**
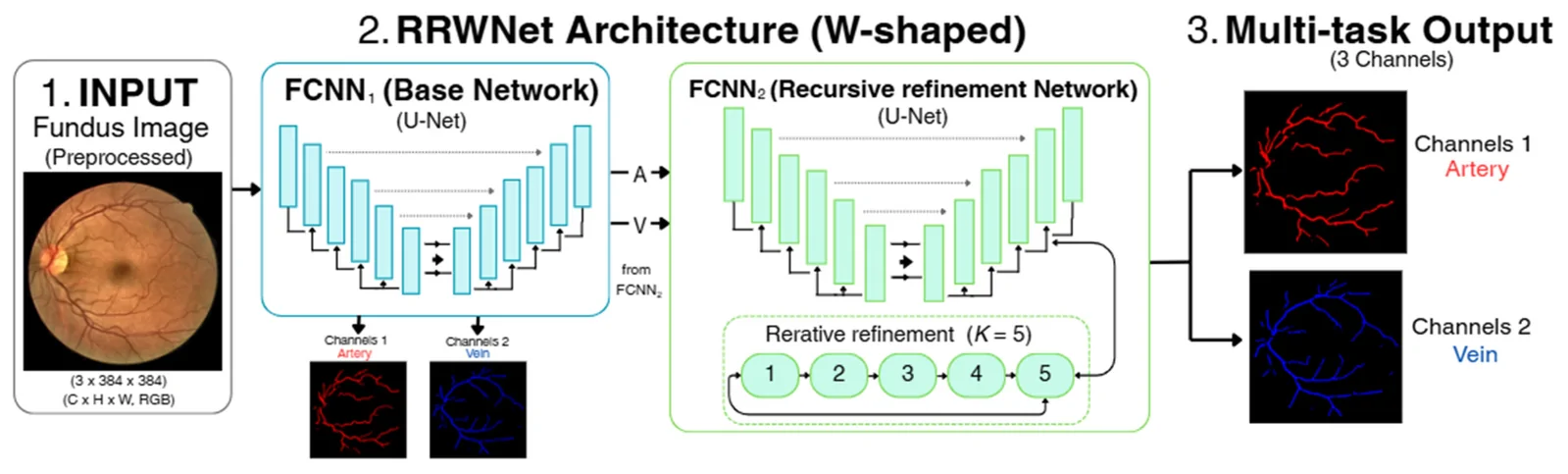
Overall architecture of the proposed RRWNet framework. Two U-Net-style FCNNs are connected in a W-shaped structure: base network FCNN_1_ produces coarse artery/vein/vessel segmentation maps, and FCNN_2_ iteratively refines artery/vein maps *K* = 5 times. Training uses a weighted sum of per-channel BCE + Dice losses; for vessel-only datasets, only the vessel-channel loss is applied. In inference post-processing, FOV masking and binarization are followed by artery/vein determination, crossing detection, and junction detection, sequentially. Dashed arrows denote the recursive refinement path, in which the predicted A/V maps are iteratively fed back into the refinement network. In the equations, the variable K is set in italics.

This study fine-tunes the model using the publicly released pre-trained weights j-morano/rrwnet-hrf by Morano et al. [19] as initialization (the full training and implementation configuration is provided in Appendix A, Table A1). The base model has 3 input channels, 3 output channels (A, V, BV), and a base channel width of 64. The recursive refinement iteration count *K* = 5 follows the default value from the original RRWNet paper [19]. All trainable parameters are retained from the base model, with only the final classification head re-trained to match the dataset distribution of this study. Figure 1 provides an overview of the complete RRWNet framework proposed in this study. FCNN_1_ (base network) produces coarse segmentation maps of arteries, veins, and vessels from fundus images, and FCNN_2_ (recursive refinement network) then takes only the artery/vein maps as input and performs *K* = 5 iterative refinements. During training, per-channel BCE + Dice losses are summed with weights (*w* = 1.0/1.5/0.5); for vessel-only datasets (DRIVE [24], CHASE_DB1 [25], HRF [23]), only the vessel-channel loss is applied. At inference, the post-processing pipeline sequentially performs FOV masking, thresholding and artery/vein determination, and crossing/junction detection.

### 3.2. Multi-Dataset Integrated Training

All eight datasets are integrated into the same training pipeline. For A/V-labeled datasets (Fundus-AVSeg [26], LES-AV [22], RITE [21], FIREFLY-Gen [20], PSEUDO_AV), the losses for all three channels—artery, vein, and vessel—are backpropagated. For vessel-only-label datasets (DRIVE [24], CHASE_DB1 [25], HRF [23]), the artery and vein channels are excluded from training. All input images are uniformly resized to 384 × 384 pixels and normalized with ImageNet statistics to maintain distributional alignment with the pre-trained weights.

### 3.3. Vessel-Only Loss Separation Strategy

To prevent A/V metric dilution caused by label heterogeneity, a strategy is introduced that branches the loss function according to the dataset tag. When the dataset tag does not belong to VESSEL_ONLY_TAGS = {DRIVE [24], CHASE_DB1 [25], HRF [23]}, the standard combined loss is applied.

*L* = *w*_0_
*ℓ*_A_ + *w*_1_
*ℓ*_V_ + *w*_2_
*ℓ*_BV_ (1)

Equation (1) defines the multi-task output loss. Here ℓ_A_, ℓ_V_, and ℓ_BV_ are the per-pixel sum of binary cross-entropy and Dice loss for the artery, vein, and vessel channels, respectively, with *w*_0_ = 1.0, *w*_1_ = 1.5, and *w*_2_ = 0.5. The higher weight for the vein channel compensates for the tendency of the stronger vein signal to overshadow artery learning. When the dataset tag belongs to VESSEL_ONLY_TAGS, the loss function is reduced as follows.

*L*_vessel-only_ = *w*_2_
*ℓ*_BV_ (2)

Equation (2) defines the reduced loss applied to vessel-only-label datasets (DRIVE, CHASE_DB1, HRF). With this branching design, incorrect artery/vein ground-truth is not forced for vessel-only datasets; instead, the model reinforces only its vessel detection capability on those images, recovering validation DSC by 0.052 and raising AUC to its highest level of 0.959.

### 3.4. Inference Post-Processing

The following post-processing steps are applied sequentially to the pixel probability maps produced by the trained model. (i) FOV masking: to suppress false positives arising from the dark border regions of fundus images, a contour-based FOV mask is extracted from the image itself and multiplied element-wise with the segmentation maps. (ii) Morphological refinement: morphological opening with a 3 × 3 kernel removes isolated point noise, and closing with a 5 × 5 kernel fills small holes within vessels. (iii) Connected-component noise removal: all connected components with area less than 50 pixels are eliminated (empirically determined through visual inspection). (iv) Artery/vein determination: within the vessel region, pixels where artery probability exceeds vein probability and is at least 0.3 are classified as artery; the remaining pixels are classified as vein. This threshold of 0.3 was empirically settled upon after validating 0.7 (an attempt to reduce background misclassification) and 0.2 (an attempt to boost recall) in initial experiments, as mean-AV DSC was highest near 0.3 compared with either extreme.

### 3.5. 8-Channel Output—Crossing and Junction Extraction

In addition to the standard artery, vein, and vessel masks and A/V composite image, the post-processing stage further produces crossing and junction maps. Crossings are obtained by computing the intersection of artery and vein masks dilated with a 5 × 5 kernel, then removing connected components with area less than 20 pixels. Junctions are detected as skeleton points on the vessel mask skeleton that have three or more neighboring pixels, then dilated with a 7 × 7 kernel for visualization. These two maps serve as source data for downstream clinical applications such as automated AVR computation and vessel branching density analysis.

### 3.6. Experimental Setup

#### 3.6.1. Dataset

The training and evaluation dataset composition of this study is summarized in Table 1.

For transparency regarding data handling, we state the following explicitly. Each dataset was divided into training and validation splits (85:15, except 95:5 for the large synthetic FIREFLY-Gen). No separate patient-level hold-out test set was isolated, and the re-inference evaluation reuses images seen during training; consequently, the reported figures reflect internal consistency and should not be read as unbiased external performance. Patient-level (group) splitting was not enforced, so fellow-eye correlation cannot be excluded. These points are treated as limitations (Section 5.3) and define our primary directions for future work. A total of eight datasets were assembled for training: five A/V-labeled (Fundus-AVSeg [26], LES-AV [22], RITE [21], FIREFLY-Gen [20], PSEUDO_AV) and three vessel-only-labeled (DRIVE [24], CHASE_DB1 [25], HRF [23]). Each dataset was split with 85% for training (95% for FIREFLY-Gen). The remaining split was used for validation. The FIREFLY-Gen dataset was shared by the original authors [20]. PSEUDO_AV is a pseudo-labeled A/V dataset generated from separate fundus images using RRWNet pre-trained weights; since model outputs rather than human annotations are used as ground truth, label reliability may be lower. This study used publicly available datasets (Fundus-AVSeg, LES-AV, RITE, DRIVE, CHASE_DB1, HRF) and a shared synthetic dataset (FIREFLY-Gen, provided by the original authors). These datasets are fully anonymized and publicly released or shared under research agreements; therefore, informed consent was waived. The study complies with the Declaration of Helsinki and relevant guidelines for research using publicly available datasets.

#### 3.6.2. Training Configuration

The Adam optimizer (*β*_1_ = 0.9, *β*_2_ = 0.999) was used with a constant learning rate of 5 × 10^−5^ for 50 epochs; the training and validation curves over all 50 epochs are shown in the training-curve figure, where the loss plateaus and validation DSC/AUC stabilize in the later epochs. Batch size was 4 and input resolution was 384 × 384 pixels. Images were normalized with ImageNet statistics (mean = [0.485, 0.456, 0.406], std = [0.229, 0.224, 0.225]) to maintain distributional alignment with the pre-trained weights. Data augmentation included random horizontal/vertical flips, 90° rotations, color/brightness jitter, and random cutout. Train/validation splits were randomized at an 85:15 ratio per dataset, except for FIREFLY-Gen (95:5 due to its large synthetic sample size). Random seed was fixed at 42 for reproducibility. All experiments were conducted on a single NVIDIA GeForce RTX 5070 Ti GPU (16 GB VRAM) with Python 3.11.9 and PyTorch 2.7.1 (CUDA 12.8).

#### 3.6.3. Evaluation Metrics

The following five metrics are reported for each of the artery, vein, and vessel channels. (i) DSC = 2·TP/(2·TP + FP + FN): overlap between segmentation and ground truth. (ii) Sensitivity Se = TP/(TP + FN): fraction of true positives detected. (iii) Specificity Sp = TN/(TN + FP): fraction of true negatives correctly predicted. (iv) PPV = TP/(TP + FP): fraction of positive predictions that are true positives. (v) AUC: discrimination performance independent of threshold. The mean-AV (mAV) metric is defined as the arithmetic mean of artery and vein values. For the four pathology subgroups (normal, AMD, glaucoma, DR) of Fundus-AVSeg, all metrics are computed and reported separately per group.

## 4. Results

### 4.1. Training Performance by Version

Validation performance comparing key changes to the training pipeline step by step is summarized in Table 2.

The baseline configuration (v3) uses five datasets: Fundus-AVSeg, LES-AV, RITE, DRIVE, and FIREFLY-Gen. v10 adds PSEUDO_AV (93 externally pseudo-labeled images) and vessel-only-label datasets CHASE_DB1 and HRF to v3 but applies A/V loss uniformly across all datasets, causing inaccurate A/V labels in vessel-only datasets to act as training signal. This caused the ep20 validation DSC of v10 to drop to 0.518—approximately 0.14 below v3 at the same point (precisely 0.660 → 0.518, ΔDSC = −0.142). This large drop suggests that label heterogeneity was actively degrading training, not merely impeding it. v11 is the final configuration incorporating the vessel-only loss separation strategy, at which the validation DSC recovers to 0.570, and AUC reaches its highest value of 0.959.

From v10 to v11, vessel-only loss separation alone recovered validation DSC by +0.052, and AUC reached 0.959—the highest among the three configurations. This result is consistent with our initial hypothesis that A/V channel losses on vessel-only datasets were actively obstructing training.

### 4.2. Re-Inference Performance by Dataset

**Figure 2:**
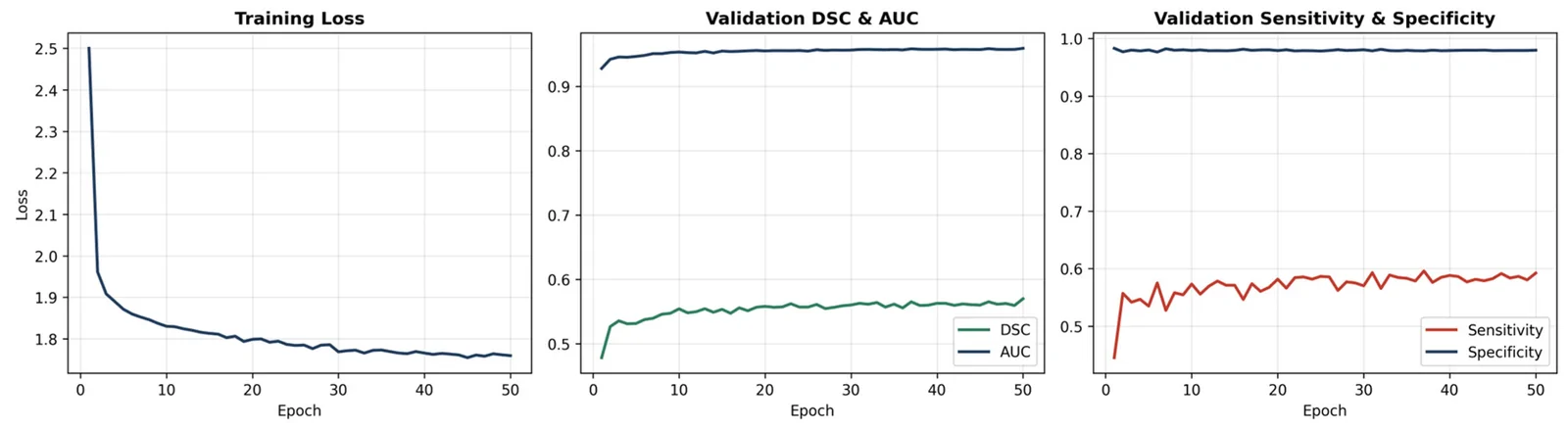
Training and validation curves over 50 epochs: (**left**) training loss; (**center**) validation Dice similarity coefficient (DSC) and area under the curve (AUC); (**right**) validation sensitivity and specificity. The loss plateaus and the validation metrics stabilize in the later epochs, indicating convergence.

We use the term re-inference to denote running the trained single-weight model over the full set of images of each dataset (including images whose splits were partly used during training); it is therefore an internal, leakage-aware assessment rather than an unbiased test-set evaluation. Results of applying the trained single-weight model to the full re-inference evaluation of four datasets (Fundus-AVSeg *n* = 100, LES-AV *n* = 99, RITE *n* = 40, PSEUDO_AV *n* = 93; 332 images total) are shown in Figure 2. Mean-AV DSC was 0.736 ± 0.036 (Fundus-AVSeg), 0.716 ± 0.030 (RITE), 0.620 ± 0.069 (LES-AV), and 0.585 ± 0.064 (PSEUDO_AV); AUC was stable at 0.96 or above across all datasets.

**Figure 3:**
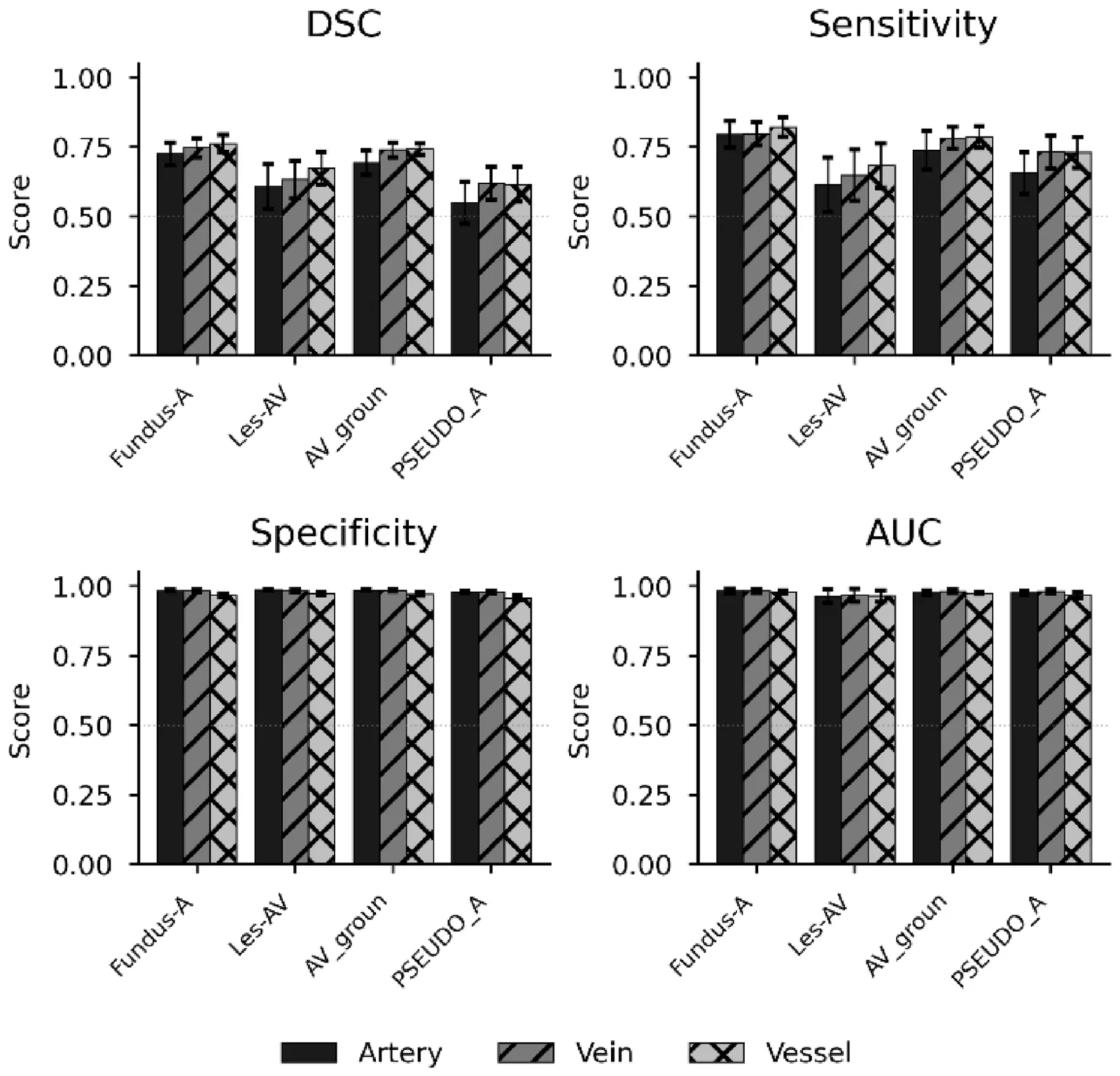
Per-dataset and per-channel performance comparison across four metrics (DSC, Se, Sp, AUC). Per-dataset and per-channel performance of the proposed model (v11) on four evaluation datasets (Fundus-AVSeg *n* = 100, LES-AV *n* = 99, RITE *n* = 40, PSEUDO_AV *n* = 93). The four panels show DSC (Dice similarity coefficient), sensitivity (Se), specificity (Sp), and AUC, respectively, with artery (solid black), vein (diagonal hatching), and vessel (crosshatched) channels displayed separately. Error bars represent image-level ± 1 standard deviation.

Figure 3 shows box plots of per-dataset DSC distributions for artery, vein, and vessel channels. Fundus-AVSeg has the narrowest IQR and highest median, making it the most stable. Extreme outliers with DSC approaching 0 are observed in some LES-AV images, attributable to the very thin color-line ground-truth masks of LES-AV, which have small pixel area. PSEUDO_AV has the lowest median and a long lower tail, directly reflecting the influence of pseudo-label noise.

### 4.3. Correlation Between Artery and Vein DSC

**Figure 4:**
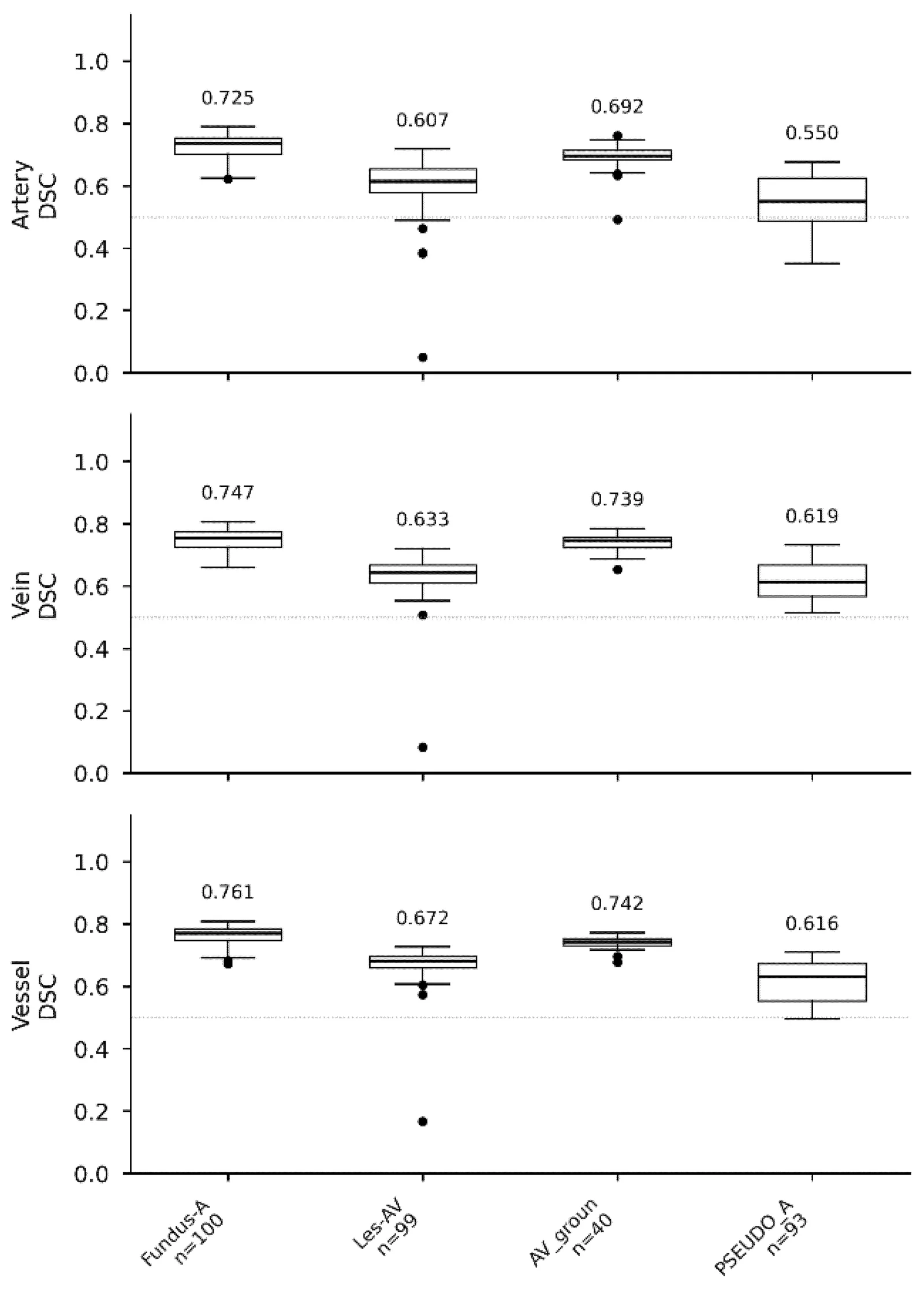
Image-level DSC distribution box plots per channel (artery, vein, vessel) for four evaluation datasets (Fundus-AVSeg *n* = 100, LES-AV *n* = 99, RITE *n* = 40, PSEUDO_AV *n* = 93). Boxes represent the IQR (Q1–Q3); lines within boxes indicate the median; whiskers extend to the max/min within 1.5 × IQR; points are outliers. Numbers above boxes indicate the image-level mean (consistent with Table 3). Dashed lines indicate the random-classification baseline (DSC = 0.5).

Figure 4 shows a scatter plot of image-level artery DSC (x-axis) versus vein DSC (y-axis). All datasets show a strong positive correlation, and most points are distributed slightly above the diagonal—indicating that vein DSC is consistently somewhat higher than artery DSC—quantitatively supporting the notion that arteries are thinner with more complex branching and thus harder to segment. Fundus-AVSeg clusters tightly at the highest positions, while PSEUDO_AV is most widely scattered.

**Figure 5:**
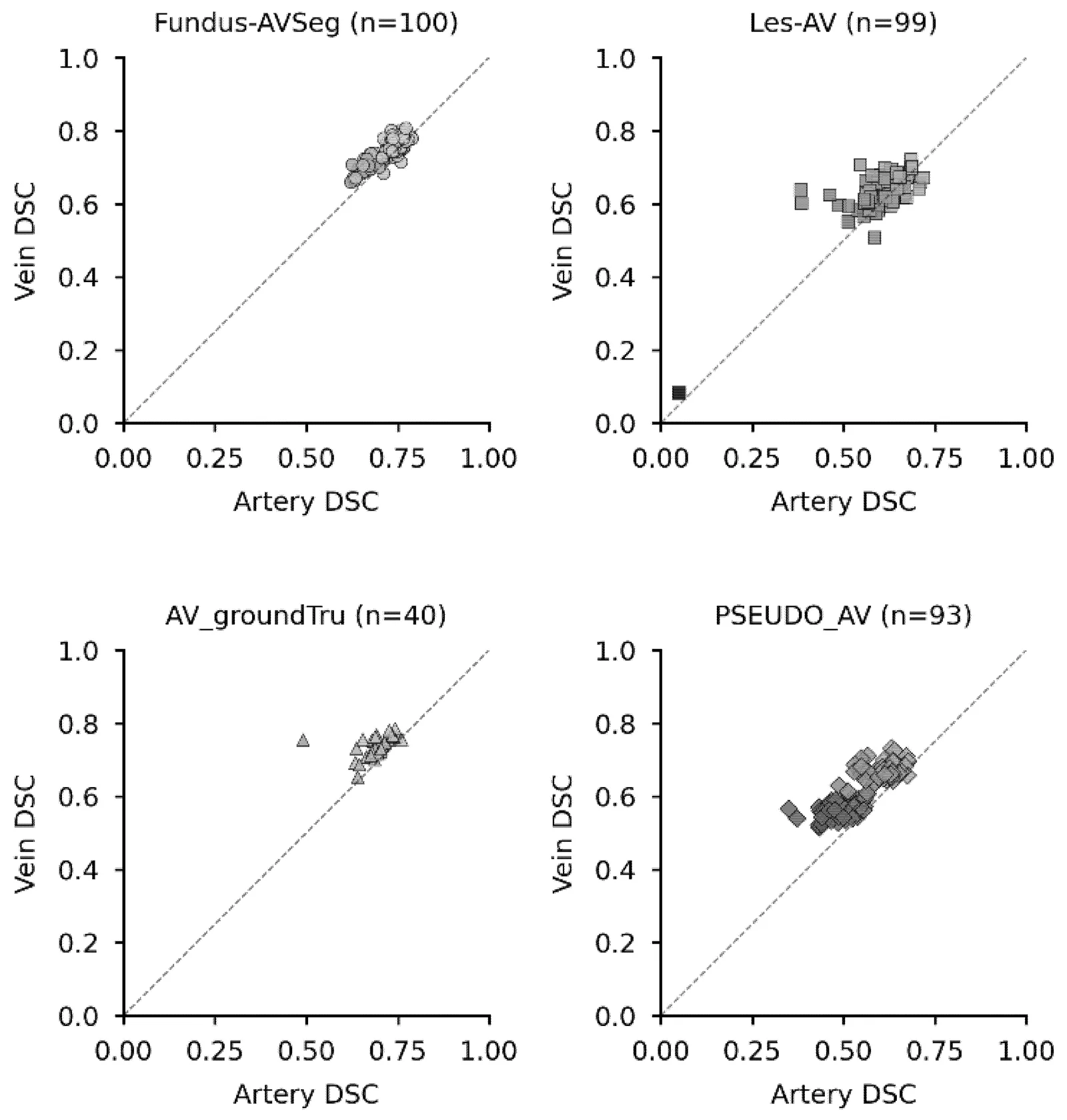
Image-level artery DSC (x-axis) vs. vein DSC (y-axis) scatter plot for four evaluation datasets. Each point corresponds to one fundus image (Fundus-AVSeg *n* = 100, LES-AV *n* = 99, RITE *n* = 40, PSEUDO_AV *n* = 93; 332 points total). The diagonal dashed line is the y = x baseline; points above it indicate images where vein was segmented better than artery.

Figure 5 shows a metric heatmap displaying all combinations of artery/vein/vessel × DSC/Se/Sp/PPV/AUC in a single view. Sp and AUC are consistently high (≥0.96) across all cells, while PPV is relatively lower. In particular, PSEUDO_AV artery PPV of 0.478 is the lowest, indicating the most frequent false-positive artery detections.

### 4.4. Quantitative Performance

Table 3 summarizes the mean and standard deviation of five metrics (DSC, Se, Sp, AUC) and mAV DSC/AUC for artery, vein, and vessel channels across four datasets.

The proposed method achieved, with a single set of weights, a four-dataset mean-AV DSC of 0.657 ± 0.085 and AUC of 0.976 ± 0.016.

Table 4 provides a direct comparison against the original RRWNet [19] trained on individual datasets. While mean-AV DSC is lower by approximately 0.10 and 0.14 on RITE and LES-AV respectively, among the four datasets across diverse domains, Fundus-AVSeg, RITE, and LES-AV all achieve mean-AV DSC above 0.62, while PSEUDO_AV remains at 0.585 due to pseudo-label noise.

### 4.5. Pathology Subgroup Analysis

**Figure 6:**
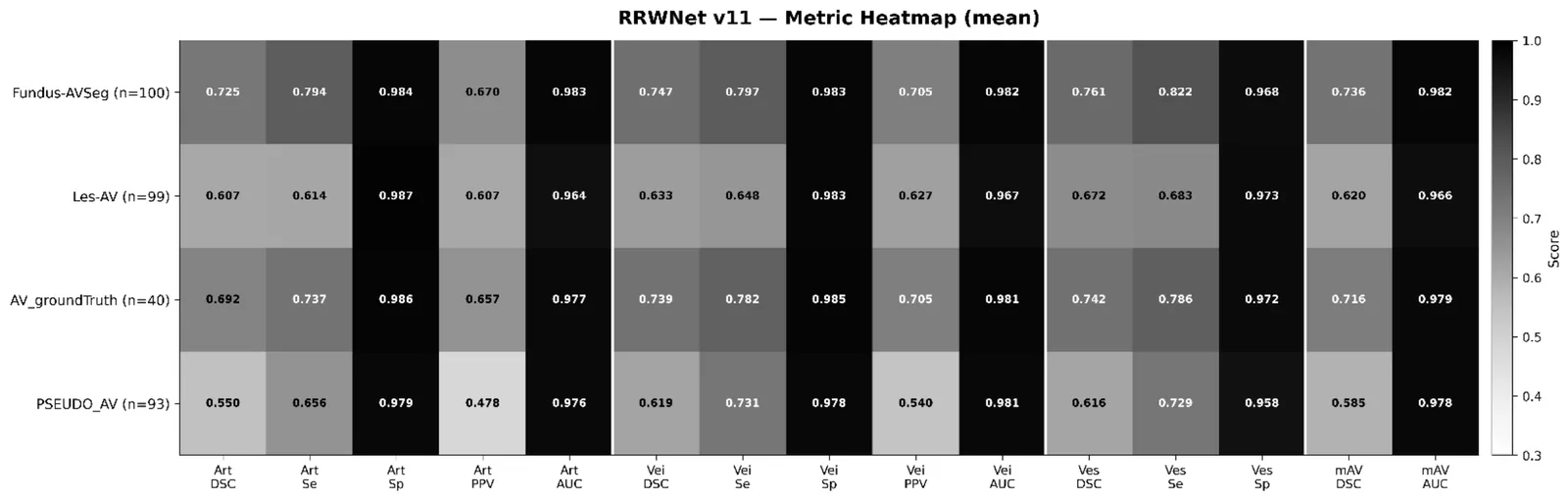
Image-level mean heatmap for 4 datasets × 3 channels (artery, vein, vessel) × 5 metrics (DSC, Se, Sp, PPV, AUC). Including 2 summary columns: mAV DSC and AUC. Cell color represents score [0.3, 1.0] as a grayscale gradient (black = high, gray = low); numerical values are shown in each cell. The vessel channel is defined as the pixel-union mask, for which PPV and AUC are not computed; those two columns are omitted. Art: artery, Vei: vein, Ves: vessel, mAV: artery–vein mean.

**Figure 7:**
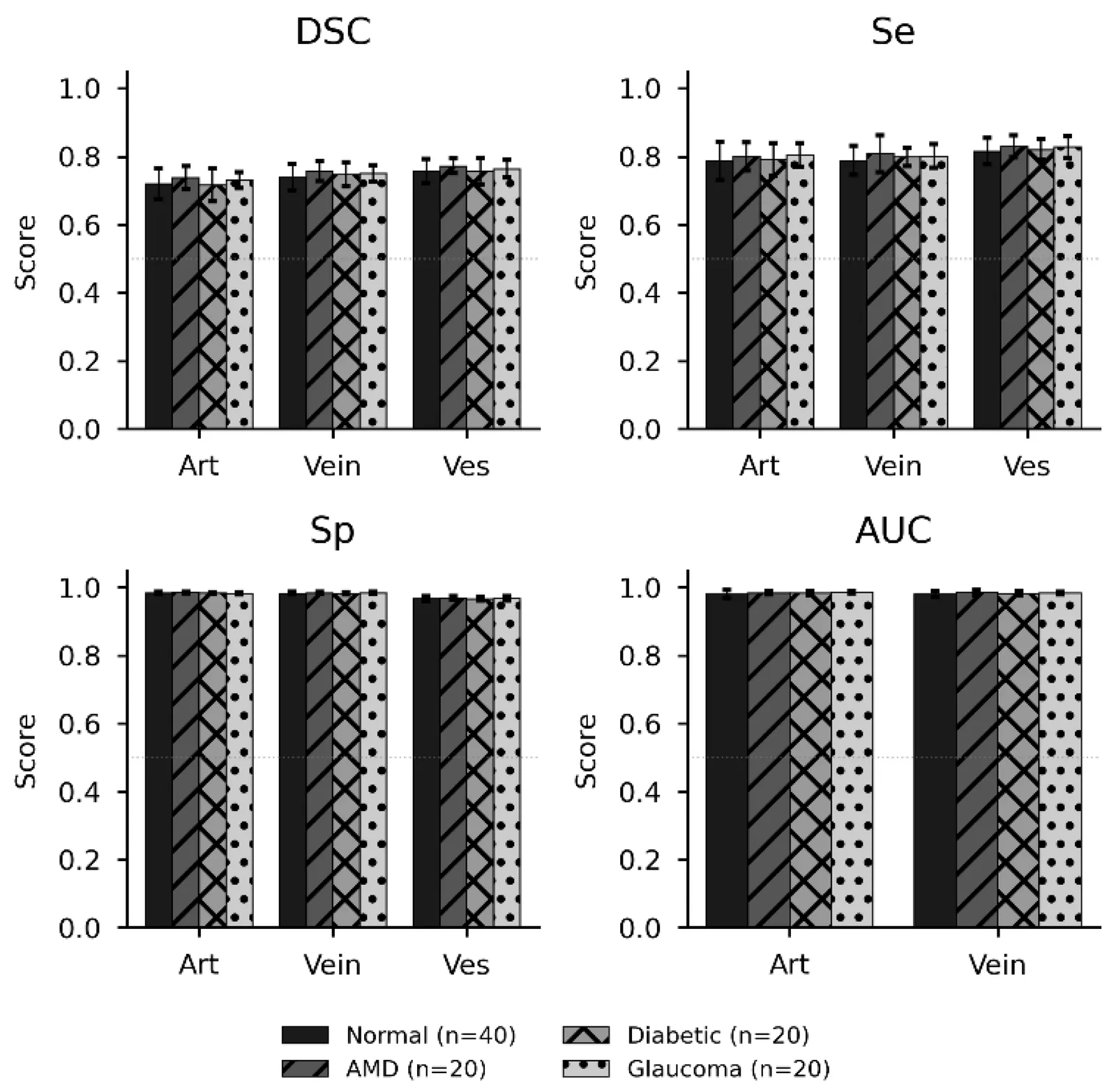
Per-pathology-subgroup, per-channel, and per-metric performance for Fundus-AVSeg (*n* = 100). The four panels show DSC, sensitivity (Se), specificity (Sp), and AUC, respectively. Within each panel, three channels—artery (Art), vein (Vein), vessel (Ves)—are placed on the x-axis, and the four pathology subgroups (normal *n* = 40, AMD *n* = 20, DR *n* = 20, glaucoma *n* = 20) are displayed as grouped bars within each channel. Error bars represent image-level ± 1 SD. PPV is addressed separately in Section 5.2 and Figure 5 and is excluded here. Dashed grid lines indicate the concentric reference scale (0.25 spacing); the near-complete overlap of the subgroup polygons indicates consistent performance across pathologies.

**Figure 8:**
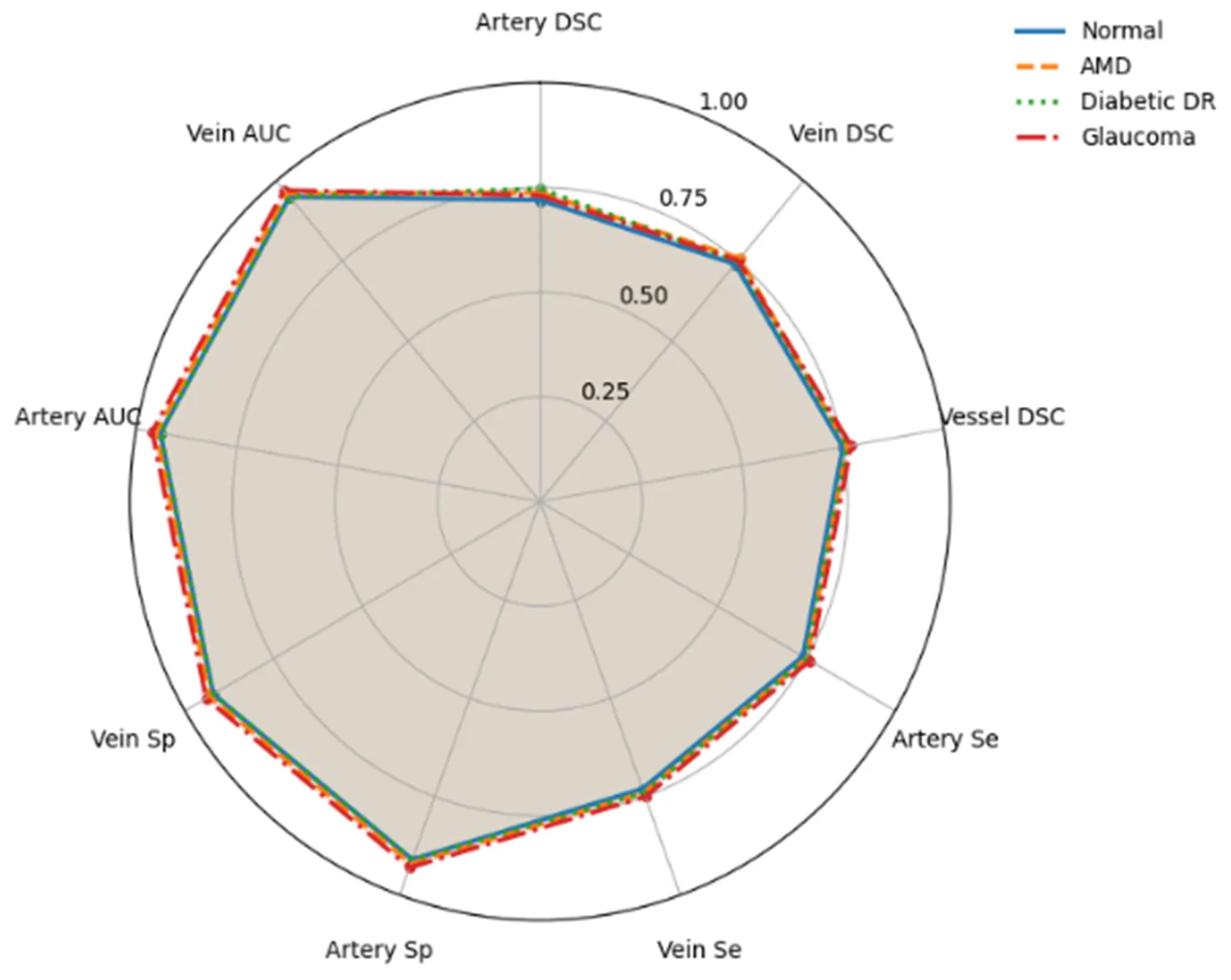
A 9-metric radar chart for four pathology subgroups (normal *n* = 40, AMD *n* = 20, DR *n* = 20, glaucoma *n* = 20). The 9 axes correspond to DSC, Se, Sp, and AUC metrics for artery, vein, and vessel channels. Each polygon represents the image-level mean of one pathology subgroup; all polygon outlines have equal thickness. The near-complete overlap of the four polygons indicates that the model operates robustly across pathology subgroups on all 9 metrics. Concentric grid spacing is 0.25.

**Figure 9:**
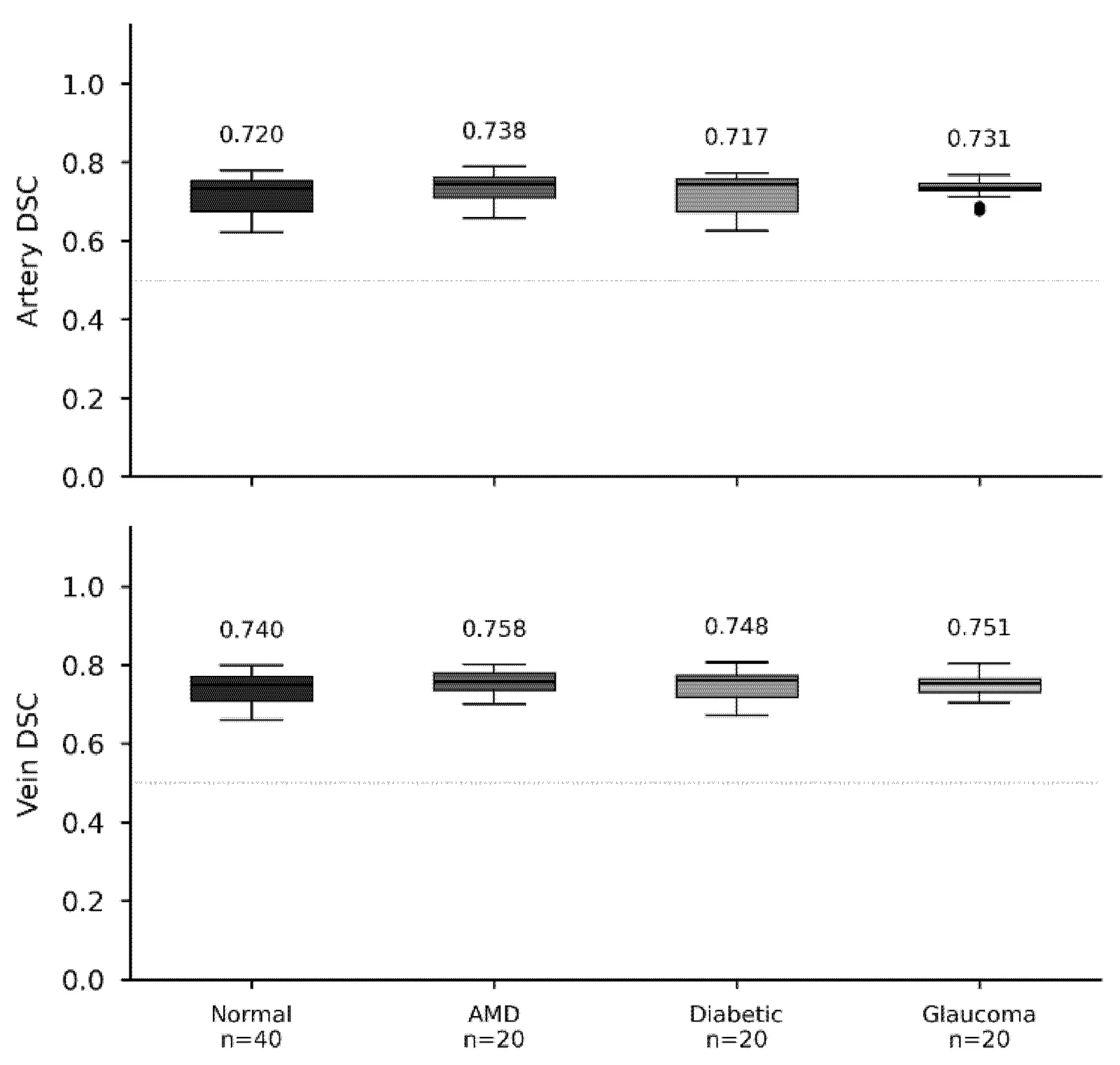
Box plots of artery DSC (**top**) and vein DSC (**bottom**) distributions by pathology subgroup for Fundus-AVSeg (*n* = 100). Boxes represent the IQR (Q1–Q3); horizontal lines within boxes indicate the median; whiskers extend to the maximum and minimum within 1.5 × IQR; points are outliers. Numbers above each box indicate the image-level arithmetic mean (consistent with Table 5). Four pathology subgroups: normal *n* = 40, AMD *n* = 20, DR *n* = 20, glaucoma *n* = 20. The vessel channel is defined as the artery–vein union and has limited additional clinical interpretive value; it is therefore omitted here. The dashed horizontal line indicates the random-classification baseline (DSC = 0.5). The dashed diagonal indicates the y = x reference line; points above it indicate images where the vein was segmented better than the artery.

Fundus-AVSeg contains four pathology labels: normal (*n* = 40), AMD (*n* = 20), glaucoma (*n* = 20), and DR (*n* = 20). Mean-AV performance for each pathology is summarized in Table 5 and Figure 6, Figure 7, Figure 8 and Figure 9.

Across subgroups, mean-AV DSC was 0.730 ± 0.041 (normal), 0.748 ± 0.028 (AMD), 0.741 ± 0.022 (glaucoma), and 0.733 ± 0.042 (DR). A Kruskal–Wallis test found no statistically significant difference among the four subgroups (mean-AV DSC H = 2.69, *p* = 0.44; artery *p* = 0.48; vein *p* = 0.43; vessel *p* = 0.49; AUC *p* = 0.36), indicating that segmentation performance was consistent across normal and pathological images rather than favoring any single subgroup. Given the modest per-group sample size within a single dataset, this subgroup analysis is reported as exploratory. The consistency observed in glaucoma images stems from the relatively small inter-patient variation in peripapillary vessel patterns. The high variability in DR is attributable to the inclusion of images with irregular lesions—including neovascularization, hemorrhages, and cotton-wool spots—that blur vessel boundaries. Failure-case analysis (Section 4) further supports a label-quality rather than pathology-driven interpretation: among the 20 lowest mean-AV DSC cases, 17 originate from PSEUDO_AV and 3 from LES-AV, whereas the highest-scoring cases are all from the expert-annotated Fundus-AVSeg set. This concentration of failures in the pseudo-labeled subset indicates that the dominant error source is annotation noise (consistent with the low artery PPV of 0.478 on PSEUDO_AV) rather than intrinsic difficulty of any pathology group. To quantify the impact of the pseudo-labeled data on the reported aggregate performance, we compared the mean-AV DSC with and without PSEUDO_AV: excluding PSEUDO_AV increased the overall mean-AV DSC from 0.657 to 0.685, and PSEUDO_AV alone yielded the lowest subset score (0.585). This indicates that pseudo-label noise depresses the aggregate metric, and that artery precision is most affected because arteries are thinner and more readily mislabeled. We therefore report PSEUDO_AV results separately and interpret them with caution; validating pseudo-labels against expert annotations is left for future work. From a clinical standpoint, the current average performance is not yet sufficient to replace expert reading; the model is positioned as an assistive tool that provides separated artery/vein masks and derived indices (e.g., AVR) for screening support. Prospective, multi-reader clinical evaluation and leakage-free external validation remain necessary before any diagnostic deployment. As a concrete step toward such validation, we have formed a clinical research group of ophthalmologists and board-certified specialists at Konyang University Hospital, with whom we plan to conduct expert-in-the-loop assessment and prospective clinical validation of the segmentation outputs and the derived indices (e.g., AVR) in future work.

### 4.6. Qualitative Results

**Figure 10:**
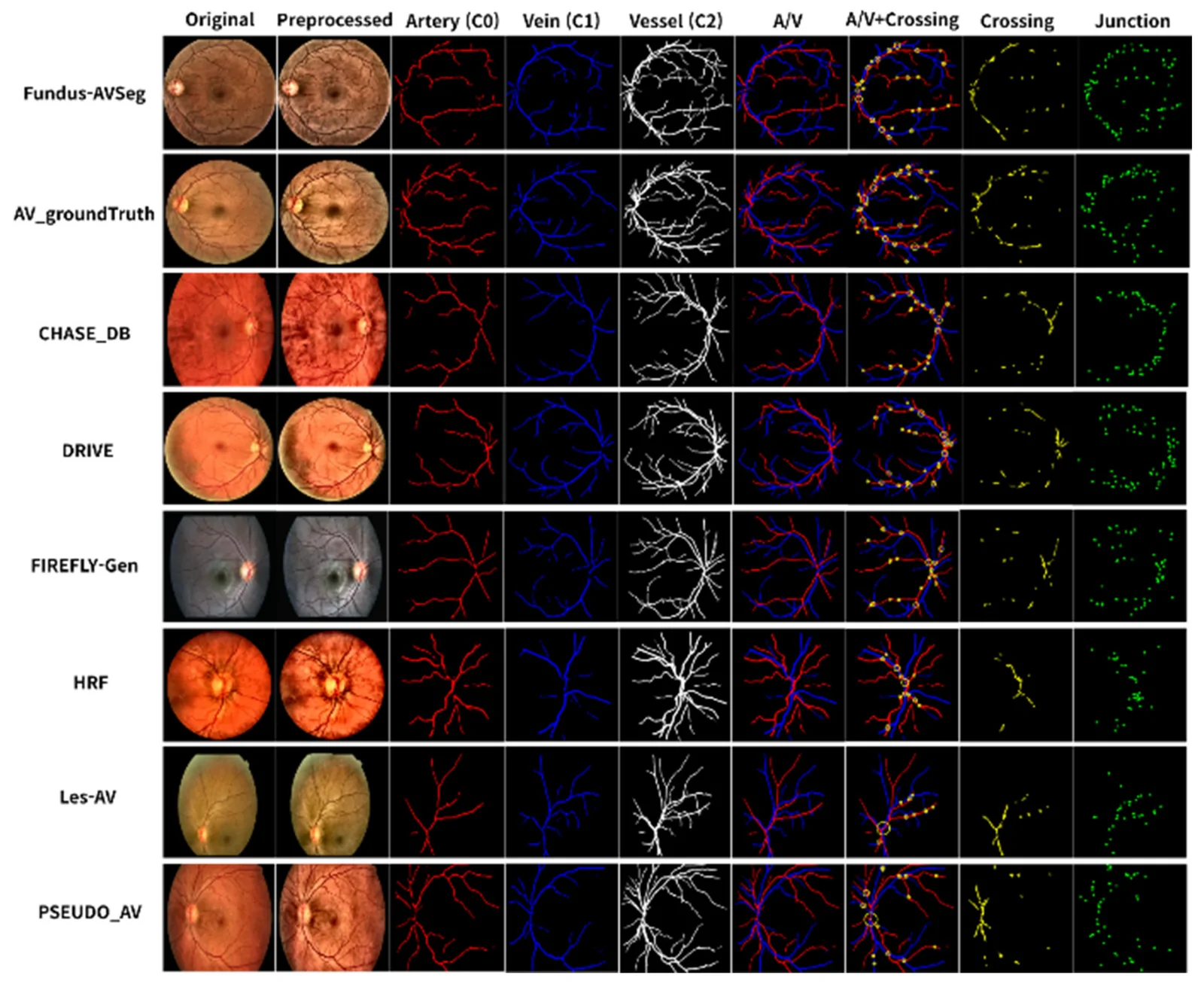
Qualitative results for one representative image from each of the eight datasets. Rows: Fundus-AVSeg, AV_groundTruth, CHASE_DB, DRIVE, FIREFLY-Gen, HRF, LES-AV, PSEUDO_AV. Columns: (1) Original—original fundus image, (2) Preprocessed—preprocessed image, (3) Artery (C0)—artery mask, (4) Vein (C1)—vein mask, (5) Vessel (C2)—vessel mask, (6) A/V—artery/vein composite, (7) A/V + Crossing—crossing overlay, (8) Crossing—crossing map, (9) Junction—junction map. The single-weight model visually produces consistent artery/vein topological structure and stable crossing/junction detections across all eight datasets.

To complement the quantitative analysis, the full model output for one representative image from each of the eight datasets is visualized in Figure 10. Each row corresponds to a dataset; each column corresponds to an output stage of the inference pipeline: (1) original fundus image, (2) preprocessed image, (3) artery mask (C0), (4) vein mask (C1), (5) vessel mask (C2), (6) A/V composite image, (7) crossing overlay, (8) crossing map only, and (9) junction map only.

Examining row by row, datasets trained with all three A/V/BV channel losses (Fundus-AVSeg, RITE, LES-AV, PSEUDO_AV, FIREFLY-Gen) show artery and vein branching patterns maintained consistently without color inversion from the optic disc to the periphery. This visually confirms that RRWNet’s recursive refinement (*K* = 5) effectively suppresses manifest classification errors even in this study’s multi-dataset training environment. Even for vessel-only-label datasets (CHASE_DB1, DRIVE, HRF), the vessel-only loss separation strategy enables stable vessel mask (C2) production; the A/V channels, although excluded from loss during training, produce plausible artery/vein segmentation through transfer of pre-trained topological information. In the HRF row, however, fine vessels tend to be segmented shorter than in other datasets, likely due to the large optic disc and uneven illumination. Crossing detections (column 8) are most dense around the optic disc, while junctions (column 9) are distributed uniformly across the entire vasculature. These qualitative patterns are broadly consistent with the per-dataset mean-AV DSC rankings reported in the quantitative results (Table 3).

## 5. Discussion

### 5.1. Trade-Off Between Multi-Dataset Applicability and Per-Dataset Peak Performance

The integrated training model of this study performs lower than single-dataset-dedicated training by mean-AV DSC 0.104 on RITE and 0.140 on LES-AV (Table 3). This is not an intrinsic limitation of integrated training but a structural cost of a single set of weights having to simultaneously absorb differences in image distribution, label definition, and resolution across eight datasets. In return, the model maintained a mean-AV AUC of 0.976 across all 332 re-inference images from the four datasets including Fundus-AVSeg, RITE, LES-AV, and PSEUDO_AV. However, since this evaluation is a re-inference over datasets partially included in training, it has limitations as a measure of fully held-out generalization performance. The lower performance on RITE and LES-AV relative to dedicated models can be viewed as a generalization tax incurred when a single set of weights must handle multiple domains simultaneously; in return, the model exhibited consistent behavior with a single set of weights across datasets from diverse institutions and pathologies. This differentiates it from single-dataset-dedicated training in that it pursues robustness-oriented modeling that is not biased toward specific dataset characteristics, and the exclusive use of publicly available datasets—with reproducibility in mind—further supports this. However, such domain-robustness superiority must be confirmed by leakage-free independent external validation and cannot be concluded from the re-inference results of this study alone.

### 5.2. PPV and False-Positive Patterns

Figure 5 shows that Sp and AUC are consistently high (≥0.96) across all cells of the heatmap, while PPV remains at approximately 0.670 for artery and 0.705 for vein. This indicates that the model fails to sufficiently suppress false positives in which background pixels are classified as vessels near the current operating threshold of 0.3. Future improvements to PPV may be achieved through: (i) raising the threshold from 0.3 to between 0.5 and 0.7; (ii) introducing focal or boundary weighting in the loss; or (iii) strengthening morphological erosion in post-processing. In particular, PSEUDO_AV artery PPV of 0.478 is a direct consequence of pseudo-label noise, indicating that the label quality of this dataset itself warrants re-evaluation.

### 5.3. Limitations

(i) The gap between validation DSC (0.570) and re-inference DSC (0.657) is a measurement artifact caused by the validation set being a ConcatDataset of validation splits from all eight datasets (5% for FIREFLY-Gen, 15% for the rest), where vessel-only dataset splits dilute the mean-AV metric. Consequently, validation DSC is a weak model-selection signal. (ii) The RITE/LES-AV figures in Table 4 are not directly comparable to the original paper [19] owing to different split configurations and are provided for reference only. (iii) At the re-inference evaluation stage, no separate hold-out test set was isolated; inference was performed over the entire dataset including splits partly used in training. This constitutes a degree of data leakage and may cause the reported DSC/AUC to somewhat overestimate true domain generalization performance. (iv) Patient-level splitting was not applied. Left- and right-eye images from the same patient may have been included simultaneously in training, validation, and re-inference splits, raising the risk that the model implicitly learned patient-specific characteristics. As patient-level split is a core requirement for evaluation reliability in medical imaging, future work will re-verify the results of this study using patient-ID-based splits. (v) No separate validation procedure was performed for label reliability of PSEUDO_AV and FIREFLY-Gen; pseudo-label noise may have affected both training signals and evaluation results. (vi) The loss curve was still descending at epoch 50, suggesting training may not have fully converged. (vii) Topology-consistency metrics (COR, the proportion of correctly connected vessels; INF, the proportion of infeasible/over-connected paths) [19] and statistical significance tests for performance differences across pathology subgroups (e.g., Kruskal–Wallis or paired Wilcoxon) were not conducted. Future work will address training extension, use of official splits with hold-out test set separation, label quality validation, COR/INF metrics, and statistical testing.

### 5.4. Clinical Implications

The fact that a single model achieves consistent performance on normal, AMD, glaucoma, and DR images suggests that it can be used in automated fundus image analysis pipelines for multi-site cohort studies or primary care settings without domain-specific retraining. The additionally produced crossing and junction maps can serve as source data for computing clinical quantitative indices such as automated AVR computation, vessel branching density analysis, and tortuosity measurement.

## 6. Conclusions

This study proposed a retinal A/V segmentation framework that uses RRWNet [19] pre-trained weights as initialization and jointly fine-tunes eight heterogeneous datasets—comprising A/V-labeled, vessel-only-labeled, and synthetic image datasets—under a single set of weights. As a key contribution, the vessel-only loss separation strategy was introduced to mitigate A/V metric dilution caused by label heterogeneity in vessel-only datasets, recovering validation DSC from 0.518 to 0.570. In re-inference evaluation across four datasets (332 images), the single-weight model achieved mean-AV DSC of 0.657 ± 0.085 and AUC of 0.976 ± 0.016. In an exploratory pathology subgroup analysis, performance did not differ significantly among normal, AMD, glaucoma, and DR images (Kruskal–Wallis, all *p* > 0.36), indicating consistent behavior across pathologies. An 8-channel output pipeline was constructed to simultaneously produce artery, vein, and vessel masks together with crossing and junction maps, providing inputs for downstream clinical applications. The largest barrier to clinical deployment is that the average re-inference performance has not yet reached the level required to replace expert reading; this model remains an assistive rather than standalone diagnostic tool. Bridging this gap will require larger expert-labeled cohorts and prospective clinical validation. We emphasize that the present evaluation is a leakage-aware internal assessment, and the reported figures should be read as internal consistency rather than generalization performance. Accordingly, our priority directions for future work directly target this limitation: (i) reconstructing the split with a strict, patient-level hold-out test set that is never seen during training, validation, or model selection, and repeating the entire experiment under patient-ID-based grouping to eliminate fellow-eye leakage; (ii) leakage-free external validation on an independent A/V dataset acquired from different institutions, devices, and populations, once a suitable external A/V-labeled resource is available; (iii) extending training beyond 100 epochs with early stopping and adopting topology-consistency metrics (COR/INF); and (iv) integrating an automated AVR computation module, which is feasible with only additional post-processing since the model already outputs separated artery/vein masks. Until (i) and (ii) are completed, we refrain from claiming domain-robustness superiority.

## Figures and Tables

**Table 1:** Dataset composition used for training and evaluation.

Dataset	Type	GT Type	# Images	Pathology	Loss Applied
Fundus-AVSeg	Real	A/V GT	100	Normal/AMD/DR/Glaucoma	A + V + BV
LES-AV [22]	Real	A/V GT	99	Normal/Glaucoma	A + V + BV
RITE [21] (DRIVE-AV [24])	Real	A/V GT	40	Normal/Mild DR	A + V + BV
FIREFLY-Gen [20]	Synthetic	A/V GT	12,000	Normal	A + V + BV
PSEUDO_AV	Pseudo-label	A/V pseudo	93	Normal	A + V + BV
DRIVE [24]	Real	BV only	40	Normal/Mild DR	BV only ★
CHASE_DB1 [25]	Real	BV only	28	Normal	BV only ★
HRF [23]	Real	BV only	45	Normal/DR/Glaucoma	BV only ★

★ VESSEL_ONLY_TAGS—vessel-channel loss only; A/V channel losses are not applied.

**Table 2:** Validation performance by training configuration (best epoch).

Version	Epoch	DSC	AUC	Se	Key Change
v3	20	0.660	0.935	0.677	5-dataset baseline
v10	20	0.518	0.928	0.533	+CHASE/HRF/PSEUDO (no loss separation)
v11	50	0.570	0.959	0.592	vessel-only loss separation

Validation performance by training configuration for four evaluation datasets (validation splits of the 8-dataset pool). v3 = 5-dataset baseline; v10 = 8 datasets without loss separation; v11 = 8 datasets with vessel-only loss separation (final configuration). All values are image-level mean-AV.

**Table 3:** Re-inference quantitative summary—mean ± SD by dataset, channel, and metric.

Metric	Fundus-AVSeg	RITE	LES-AV	PSEUDO_AV
***n***	100	40	99	93
Artery DSC	0.7251 ± 0.041	0.6919 ± 0.043	0.6068 ± 0.080	0.5502 ± 0.075
Artery Se	0.7943 ± 0.049	0.7371 ± 0.069	0.6144 ± 0.098	0.6558 ± 0.074
Artery Sp	0.9835 ± 0.003	0.9859 ± 0.003	0.9867 ± 0.002	0.9792 ± 0.005
Artery AUC	0.9826 ± 0.008	0.9771 ± 0.009	0.9643 ± 0.025	0.9763 ± 0.007
Vein DSC	0.7471 ± 0.034	0.7392 ± 0.027	0.6330 ± 0.067	0.6194 ± 0.059
Vein Se	0.7973 ± 0.042	0.7817 ± 0.039	0.6481 ± 0.091	0.7313 ± 0.058
Vein Sp	0.9831 ± 0.004	0.9852 ± 0.004	0.9833 ± 0.004	0.9783 ± 0.005
Vein AUC	0.9824 ± 0.007	0.9813 ± 0.005	0.9668 ± 0.023	0.9806 ± 0.007
Vessel DSC	0.7614 ± 0.033	0.7416 ± 0.019	0.6725 ± 0.059	0.6156 ± 0.063
**mAV DSC**	**0.7361 ± 0.036**	0.7155 ± 0.030	0.6199 ± 0.069	0.5848 ± 0.064
**mAV AUC**	**0.9825 ± 0.007**	0.9792 ± 0.006	0.9656 ± 0.024	0.9784 ± 0.006

All values are image-level mean ± 1 SD. Bold: best per row across four datasets. mAV: arithmetic mean of artery and vein channels. *n*: number of images. Table values are per-dataset macro-averages; the image-level weighted mean across all 332 images of four datasets is mAV DSC = 0.657 ± 0.085 and mAV AUC = 0.976 ± 0.016, reported separately from the macro-average in the Abstract and Section 5.1. Sp = specificity, PPV = positive predictive value, AUC = area under the ROC curve.

**Table 4:** Comparison against original RRWNet [19] single-dataset-dedicated training.

Method	Eval Set	Artery DSC	Vein DSC	mAV DSC	Training Config
RRWNet [19]	RITE	0.810	0.830	0.820	Single RITE
RRWNet [19]	LES-AV [22]	0.750	0.770	0.760	Single LES-AV
**This Study**	RITE	0.692	0.739	0.716	8-dataset integrated
**This Study**	LES-AV [22]	0.607	0.633	0.620	8-dataset integrated
**This Study**	Fundus-AVSeg	0.725	0.747	0.736	8-dataset integrated (new)

All values are image-level mean-AV DSC means. Bold: rows from this study. RRWNet [19] values are from the original paper; no baseline exists for Fundus-AVSeg as it is newly introduced here. See Table 1 for sample sizes and label composition.

**Table 5:** Mean-AV performance by pathology subgroup in Fundus-AVSeg, with 95% confidence intervals (CI). A Kruskal–Wallis test found no significant difference among the four subgroups (mean-AV DSC H = 2.69, *p* = 0.44); exploratory given the modest sample sizes.

Pathology	*n*	Artery DSC	Vein DSC	mAV DSC	mAV AUC	95% CI (mAV)
**AMD**	20	0.738 ± 0.034	0.758 ± 0.029	0.748 ± 0.028	0.985 ± 0.004	[0.735, 0.760]
**Glaucoma**	20	0.731 ± 0.023	0.751 ± 0.025	0.741 ± 0.022	0.984 ± 0.002	[0.730, 0.750]
**DR**	20	0.717 ± 0.049	0.748 ± 0.036	0.733 ± 0.042	0.983 ± 0.005	[0.713, 0.750]
**Normal**	40	0.720 ± 0.045	0.740 ± 0.038	0.730 ± 0.041	0.981 ± 0.009	[0.717, 0.742]

All values are image-level mean ± 1 SD. AMD: age-related macular degeneration; DR: diabetic retinopathy. The normal group (*n* = 40) differs in sample size from the other three groups (*n* = 20 each); note the weight difference when making direct mean comparisons.

## Data Availability

The public datasets analyzed in this study are available from their original sources: Fundus-AVSeg [26], LES-AV [22], RITE/DRIVE-AV [21], DRIVE [24], CHASE_DB1 [25], and HRF [23]. The FIREFLY-Gen synthetic dataset was provided by the authors of [20]. The pre-trained RRWNet weights (j-morano/rrwnet-hrf) are publicly available [19]. Code and trained model weights are available from the corresponding author upon reasonable request.

## Abbreviations

The following abbreviations are used in this manuscript:

A/V	Artery/Vein
AMD	Age-related Macular Degeneration
AUC	Area Under the ROC Curve
AVR	Arteriolar-to-Venular Ratio
BV	Blood Vessel
CC	Connected Component
DR	Diabetic Retinopathy
DSC	Dice Similarity Coefficient
FCNN	Fully Convolutional Neural Network
FOV	Field of View
GT	Ground Truth
PPV	Positive Predictive Value
RRWNet	Recursive Refinement W-Net
Se	Sensitivity
Sp	Specificity

## Appendix A. Training and Implementation Details

For full reproducibility, the complete training and implementation configuration of the proposed framework is summarized in Table A1. All values correspond to the final adopted configuration (v11) described in Section 3, and the inference post-processing pipeline is described in Section 3.6.

**Table A1:** Training and implementation configuration of the proposed framework.

Setting	Value
Backbone	Recursive Refinement W-Net (RRWNet); W-shaped FCNN with two subnetworks
Input/output channels	3/3 (artery, vein, vessel)
Base channel width	64
Recursive refinement iterations (*K*)	5
Initialization	RRWNet weights pre-trained on HRF (j-morano/rrwnet-hrf)
Optimizer	Adam (*β*_1_ = 0.9, *β*_2_ = 0.999)
Learning rate	5 × 10^−5^ (constant)
Batch size	4
Input resolution	384 × 384 pixels
Epochs	50
Normalization	ImageNet (mean [0.485, 0.456, 0.406]; std [0.229, 0.224, 0.225])
Augmentation	Random horizontal/vertical flip, 90° rotation, color/brightness jitter, cutout
Loss function	Per-channel binary cross-entropy + Dice
Loss weights (artery/vein/vessel)	1.0/1.5/0.5
Vessel-only datasets	Vessel-channel loss only (DRIVE, CHASE_DB1, HRF)
Post-processing	FOV masking; morphological opening (3 × 3) and closing (5 × 5); connected-component removal < 50 px; A/V threshold 0.3
Train/validation split	85:15 per dataset (FIREFLY-Gen 95:5)
Random seed	42
Hardware	NVIDIA GeForce RTX 5070 Ti GPU (16 GB VRAM)
Framework	Python 3.11.9, PyTorch 2.7.1 (CUDA 12.8)
